# Heterogeneity in maternal mRNAs within clutches of eggs in response to thermal stress during the embryonic stage

**DOI:** 10.1186/s12862-024-02203-8

**Published:** 2024-02-12

**Authors:** Atsuko Sato, Yukie Mihirogi, Christine Wood, Yutaka Suzuki, Manuela Truebano, John Bishop

**Affiliations:** 1 Department of Biology, Ochanomizu University, Otsuka, Bunkyo-Ku, Tokyo, 112-8610 Japan; 2 Marine Biological Association of the UK, The Laboratory, Citadel Hill, Plymouth, PL1 2PB UK; 3 Graduate School of Frontier Sciences, University of Tokyo, Kashiwano-Ha, Chiba, 277-8561 Japan; 4 Marine Biology and Ecology Research Center, School of Biological and Marine Sciences, Plymouth University, Drake Circus, Plymouth, PL4 8AA UK; 5 Human Life Innovation Center, Ochanomizu University, Otsuka, Bunkyo-Ku, Tokyo, 112-8610 Japan; 6 Graduate School of Life Sciences, Tohoku University, 6-3, Aramaki Aza Aoba, Aoba-Ku, Sendai, 980-8578 Japan

**Keywords:** Sibling species, Hybrid, Heat stress, Variation, Bet-hedging, Single-egg sequencing, Genotype-to-phenotype map

## Abstract

**Background:**

The origin of variation is of central interest in evolutionary biology. Maternal mRNAs govern early embryogenesis in many animal species, and we investigated the possibility that heterogeneity in maternal mRNA provisioning of eggs can be modulated by environmental stimuli.

**Results:**

We employed two sibling species of the ascidian *Ciona*, called here types A and B, that are adapted to different temperature regimes and can be hybridized. Previous study showed that hybrids using type B eggs had higher susceptibility to thermal stress than hybrids using type A eggs. We conducted transcriptome analyses of multiple single eggs from crosses using eggs of the different species to compare the effects of maternal thermal stress on heterogeneity in egg provisioning, and followed the effects across generations. We found overall decreases of heterogeneity of egg maternal mRNAs associated with maternal thermal stress. When the eggs produced by the F1 AB generation were crossed with type B sperm and the progeny (‘ABB’ generation) reared unstressed until maturation, the overall heterogeneity of the eggs produced was greater in a clutch from an individual with a heat-stressed mother compared to one from a non-heat-stressed mother. By examining individual genes, we found no consistent overall effect of thermal stress on heterogeneity of expression in genes involved in developmental buffering. In contrast, heterogeneity of expression in signaling molecules was directly affected by thermal stress.

**Conclusions:**

Due to the absence of batch replicates and variation in the number of reads obtained, our conclusions are very limited. However, contrary to the predictions of bet-hedging, the results suggest that maternal thermal stress at the embryo stage is associated with reduced heterogeneity of maternal mRNA provision in the eggs subsequently produced by the stressed individual, but there is then a large increase in heterogeneity in eggs of the next generation, although itself unstressed. Despite its limitations, our study presents a proof of concept, identifying a model system, experimental approach and analytical techniques capable of providing a significant advance in understanding the impact of maternal environment on developmental heterogeneity.

**Supplementary Information:**

The online version contains supplementary material available at 10.1186/s12862-024-02203-8.

## Background

Variation, or heterogeneity, is the raw material of evolutionary innovations [1–4], and the importance of environmentally induced variation during the evolutionary process is well recognized [2]. Besides genetic variation, there have been a number of hypotheses proposing the evolutionary importance of environmental influence in producing heterogeneity without initial alteration of the genotype, such as the Baldwin effect, genetic assimilation and phenotypic accommodation [2, 5]. Waddington famously proposed that development is buffered against environmental as well as genetic variation and canalized into a single path, which he called developmental buffering. He also proposed that, when development faces a drastic environmental change, previously cryptic variation may be revealed in the form of new developmental paths, which may be fixed in a population after many generations as morphological evolution [5]. The so-called bet-hedging hypothesis, in which environmental fluctuation induces increased phenotypic heterogeneity that raises overall fitness by allowing at least some of the offspring to survive, also highlights the importance of environmentally induced variation in evolution [6–10].

Impact of maternal environment has been documented for maternal provisioning of hormones [11, 12] and egg composition [13]. A recent study also showed that maternal age affects maternal mRNA composition in mammalian eggs [14]. Maternal mRNAs are important in many animal species in shaping early embryogenesis before zygotic transcription starts [15]. Accumulating evidence suggests that maternal provisioning is important in developmental buffering, which stabilizes organismal development in the presence of thermal stress in several aquatic organisms, such as sea urchins [16], ascidians [17] and fish [18, 19]. It has been hypothesized that, by altering maternal provisioning, the maternal environment might affect the buffering level of siblings [20]. Therefore, examining changes in maternal provisioning induced by environmental stress is important in predicting future changes in the level of developmental buffering as well as developmental paths. However, the impact of maternal environment on the heterogeneity of maternal mRNAs has not been investigated. This is critical to understanding how and whether environment affects development in the next generation, providing a possible mechanism of environmentally induced evolutionary change.

We addressed the question of whether heterogeneity of maternal mRNAs in eggs is induced by heat stress using sibling species of ascidian, *Ciona intestinalis* type A (formally called *Ciona robusta*) and type B (formally called *C. intestinalis*) [21], which are adapted to different thermal environments [17]. *C. intestinalis* is a hermaphrodite invertebrate chordate, and has been a major chordate model in developmental biology for over a century given its wide availability in all oceans. However, recent studies revealed that *C. intestinalis* is in fact two inter-fertile sibling species showing different levels of thermal susceptibility [22–25]. We fertilized clutches of eggs from the two sibling species with sperm of one of the species, treated half the embryos with thermal stress at late neurula to early tailbud stage, reared them to mature adults, and compared the between-egg variation of maternal mRNAs in the eggs produced by stressed and un-stressed individuals using single-egg mRNA-seq. We stressed the neurula to early tailbud stage embryonic development, the stage at which all the characteristics of the chordate body plan appear, hence relevant to understanding evolution of the chordate body plan [25]. Our data suggested that exposure to thermal stress during early life (embryogenesis) altered variation of maternal mRNA in eggs produced at maturity, overall variation apparently being reduced in the first generation. At individual gene level, we found that heterogeneity of the two signaling molecules, namely *hh* and MEGF11 were affected by thermal stress, whereas we did not find any of the developmental buffering genes affected by thermal stress. Our study suggests the potential for alteration of development by changing environmental conditions, which might be a source of evolution of body plan.

## Results

### Maternal mRNAs in eggs are affected by maternal heat stress

**Fig. 1:**
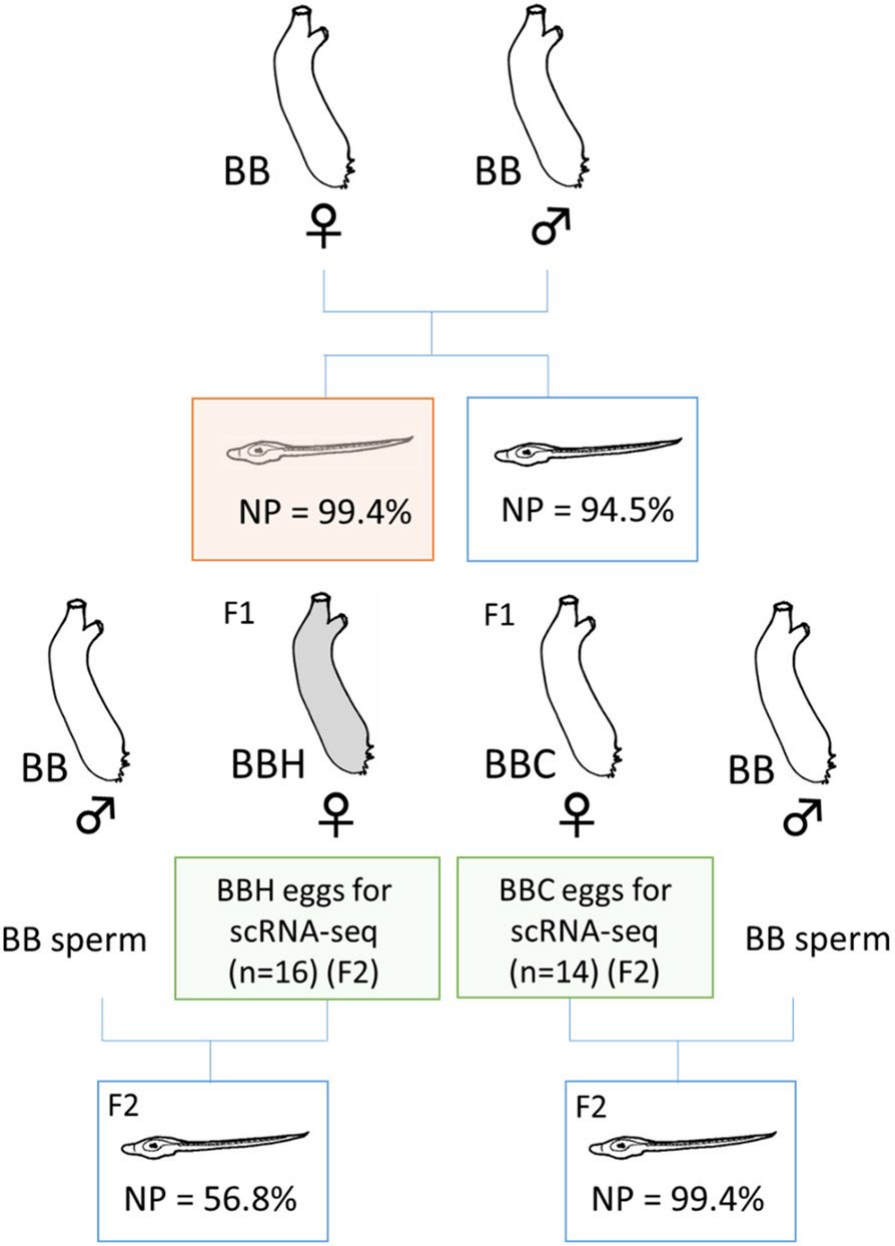
Design of experiment using a type B conspecific cross. Gametes were collected by dissection from Type B specimens taken from the wild, and sperm of one individual used to fertile eggs of another. At 8 h post fertilization, half of the embryos were exposed to 26.5 ºC for 1 h and then reared at 17 ºC until they settled, metamorphosed and started feeding, whereas the other half were reared at 17 ºC without the temperature shock. Juveniles of both batches were then transferred to a marina and cultured side-by-side. After maturing in the marina, oocytes were collected from one heat-shocked and one non-shocked (control) individual for single-cell sequencing (scRNA-seq). NP, proportion of juveniles showing normal development at hatching. A red square indicates NP after heat shock, and a white square indicates without thermal stress

Previous studies have recorded between-cell or between-individual variation in expression of genes in yeast and *Caenorhabditis elegans* [26–28], but environmentally induced heterogeneity in maternal mRNAs has not been studied. Maternal mRNAs are critical in the early embryogenesis in many animal species including *C. intestinalis*. We first set up experiments to observe environmentally induced heterogeneity in maternal mRNAs using previously determined stress criteria in which *Ciona intestinalis* (type B) is susceptible to 1 h thermal stress above 26.5 °C at 8 h post fertilization at 17 °C (late neurula stage). We exposed a batch of embryos from type B conspecific cross (BB) to thermal stress (26.5 °C for 1 h) at late neurula stage, and then transferred them back to 17 °C until hatching (at c. 24 h) and allowed them to settle. After exposure to thermal stress, some larvae hatched with deformation in the tail, notochord, or central nervous system, and some embryos did not even hatch. We counted the number of larvae that hatched normally without these deformations. The proportion of normal development at hatching was 99.4% in control embryos (‘BBC’) and 94.5% in heat-treated embryos (‘BBH’), suggesting that thermal stress caused only limited differential mortality in this experiment (Fig. 1). After settlement, we reared the progeny under field conditions in a marina, where they were all exposed to the same natural environmental conditions.

**Fig. 2:**
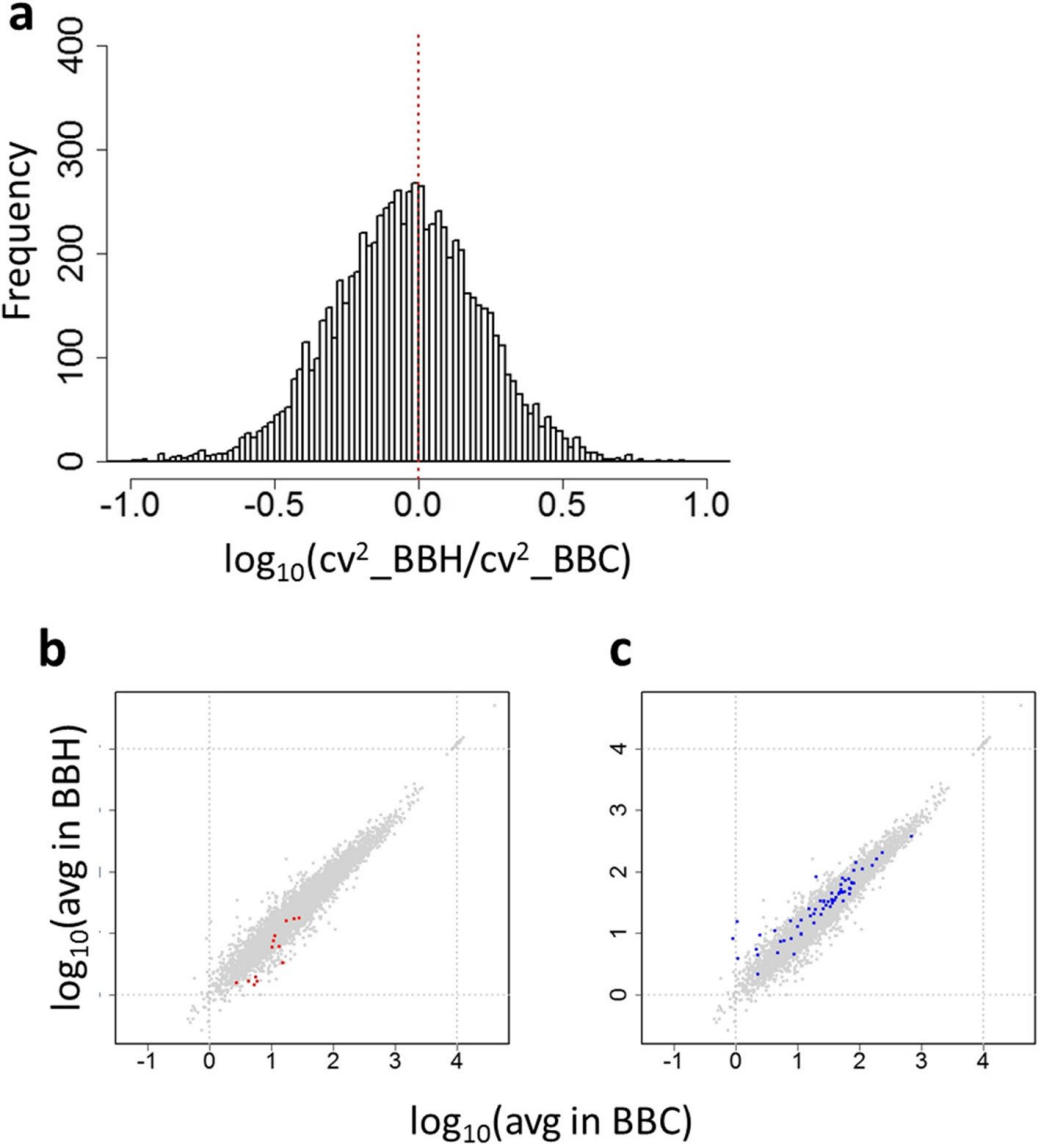
Impact of maternal embryonic heat stress on the maternally derived egg mRNAs from the type B conspecific cross. **a** Frequency of values of log_10_(CV^2^BBH/CV^2^BBC) of gene models in the eggs from heat-treated and control mothers. **b**, **c** Distribution of expression of genes showing a more than five-fold increase (shown in red) (**b**) or decrease (shown in blue) (**c**) in CV^2^ between eggs from heat-treated and control mothers. X-axis shows average expression level (TPM) in eggs from control (C) mother, and y-axis average expression level (TPM) from heat-shocked (H) mother in each batch. BBC (*n* = 14 eggs); BBH (*n* = 16 eggs). Note that genes showing a five-fold increase or (particularly) decrease of CV^2^ were spread out, and not limited to genes with low expression values

We isolated multiple single eggs from a mature BBH adult and from a mature BBC adult and compared maternal mRNA composition at single-egg level via scRNA-seq. The overall squared coefficient of variance (CV^2^) was slightly reduced following thermal stress (mean value of log_10_(CV^2^H/CV^2^C) = -0.04) (Fig. 2a-c; ANCOVA test, estimate -0.0014, t-value -7.25, *P* < 4.08e-13), decreasing in 56% of the gene models (4317 out of 7732) following maternal thermal stress, and increasing in the others. We found a significant interaction between gene model and environmental stimulus compared to a null hypothesis of non-interaction (Bartlett test, *P* < 2.2e^−16^). We also found that, following fertilization of their eggs with BB sperm, progeny of the BBH adult underwent a higher proportion of normal development (99.4%) than progeny of the BBC adult (56.8%). (Fisher’s exact test, *P* < 2.2e-16).

### Impact of maternally inherited buffering levels on mRNA variation

**Fig. 3:**
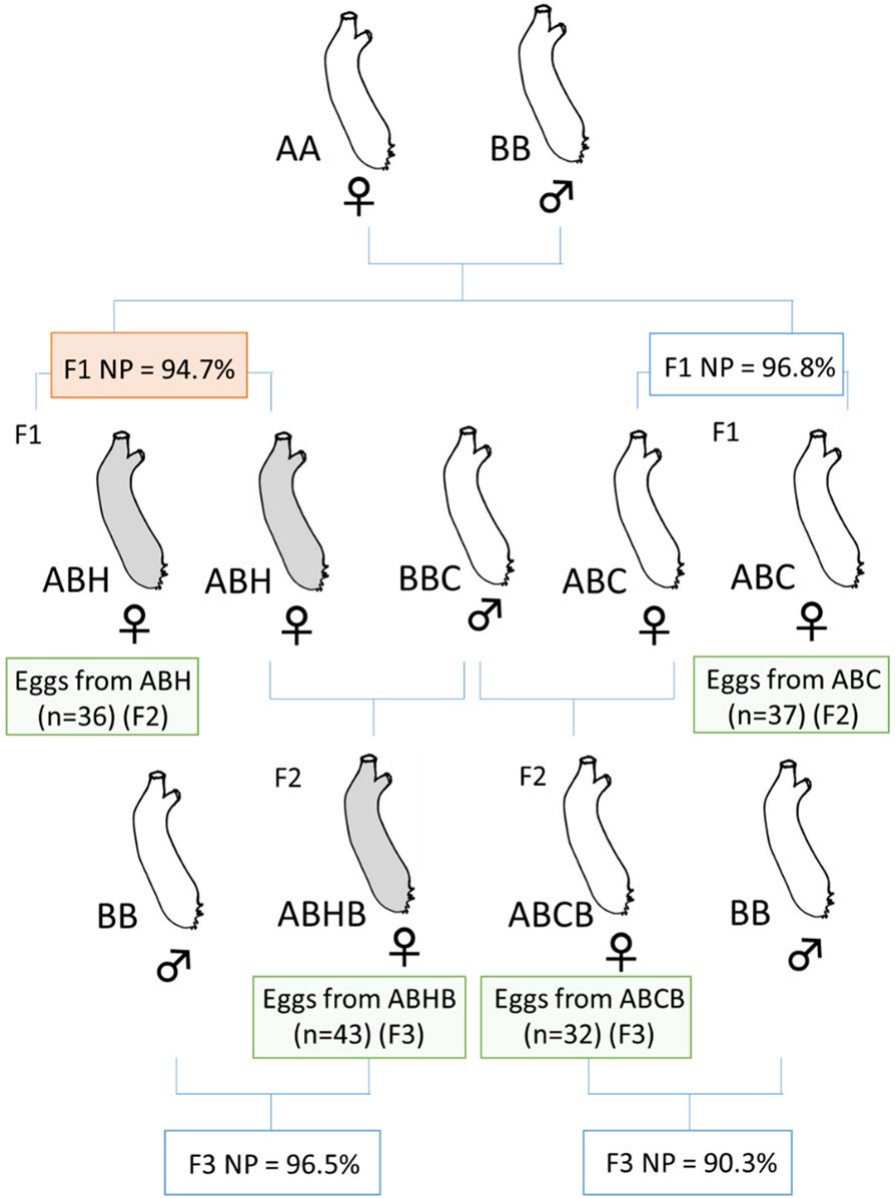
Experimental design of the crosses using type A eggs. A Type A specimen and a type B specimen, both wild-caught, were dissected, and A eggs were fertilized with B sperm. At 8 h post fertilization, half of the embryos were heat treated at 26.5 ºC for 1 h and then reared at 17 °C until they settled, metamorphosed and started feeding, whereas others were reared at 17 °C without heat shock. F1 juveniles of both batches were transferred to a marina and cultured side-by-side. When they matured, eggs were collected from one heat-shocked individual (ABH) and one non-shocked individual (ABC). Some eggs were subjected to scRNA-seq while others from the same clutches were crossed with sperm from another laboratory-reared type B specimen (BBC). Embryos were cultured without heat treatment until they matured (F2: ABCB and ABHB). Eggs from mature F2 animals were collected and subjected to scRNA-seq while others were crossed with a type B specimen taken from the wild. The resulting F3 embryos were examined to determine the proportion of normal development after hatching without heat treatment. Green rectangles show eggs collected for single-egg sequencing analyses. NP, proportion of normal development at hatching. Red square means NP after heat treatment, and white square without heat treatment

**Fig. 4:**
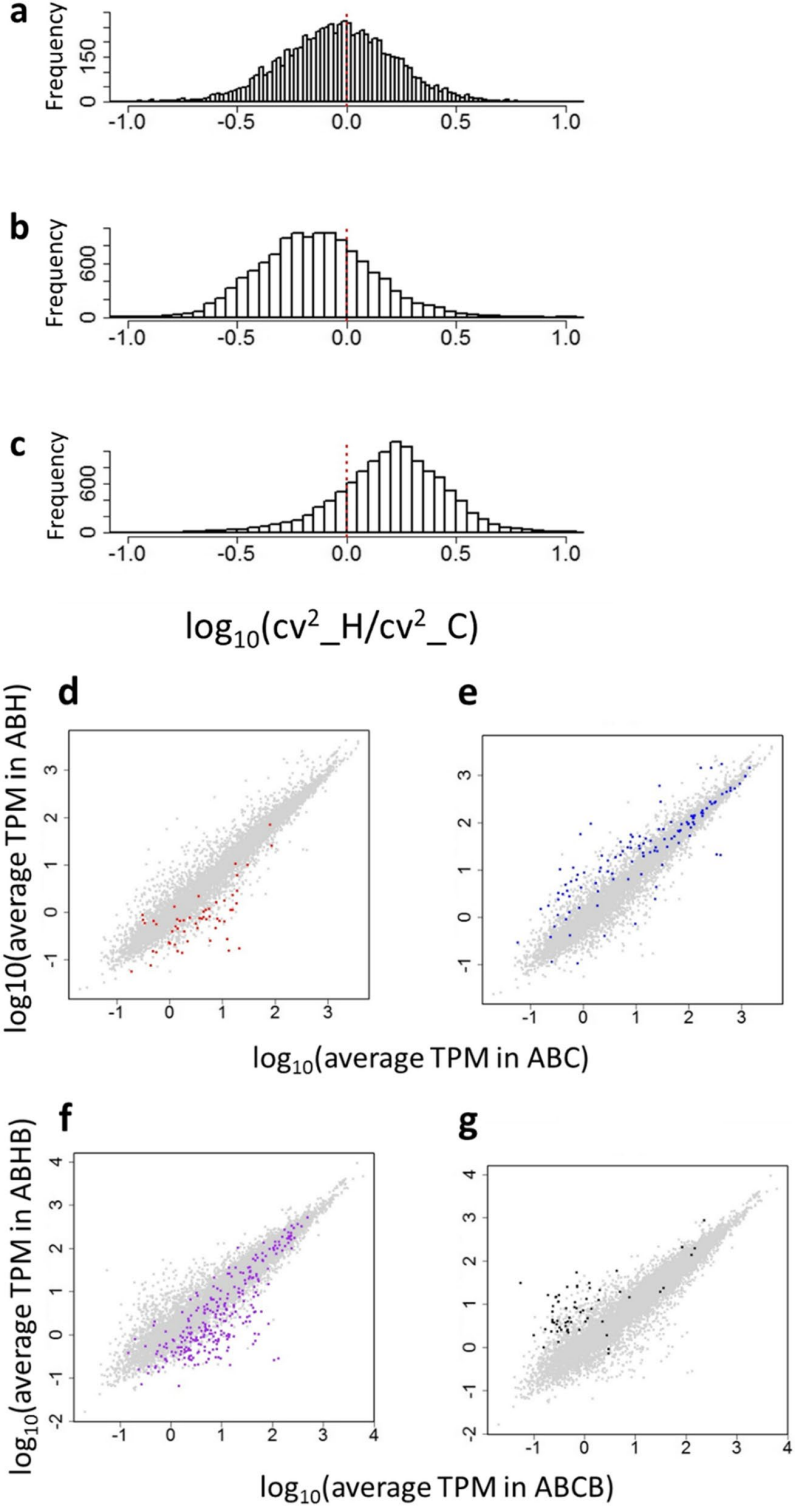
Differences in variance of expression of gene models in the eggs from different crosses in relation to maternal embryonic heat stress. Frequency of log_10_(CV^2^_H/CV^2^_C) values of gene models in eggs from individuals heat treated as embryos (H) divided by values from control individuals (C) in (**a**), BB conspecific cross, and (**b**), AB hybrid cross. In (**c**), ABB cross, the heat shock (H) had been administered to the embryo that became the AB mother of the individual producing the eggs. For each gene model the value of eggs from heat stressed individuals was divided by the corresponding value of the eggs from the same lineage but without heat shock. Red dotted lines indicate a log ratio of zero (CV^2^ values equal). **d**-**g** Distribution of genes (coloured dots) showing more than five times increase (shown in red in d, in purple in f) or decrease (shown in blue in e, shown in dark green in g) in CV^2^ between eggs from a heat-treated and a control individual in AB (**d**, **e**) or between eggs from an individual with a heat-treated mother and one with a control mother in ABB (**f**-**g**). X-axis shows average expression level (TPM) in eggs from control (C) mother, and y-axis average expression level (TPM) from heat-shocked (H) mother in each batch. Note that the genes showing a five-fold increase or decrease of CV.^2^ were not limited to genes with low expression levels. ABC (*n* = 37 eggs); ABH (*n* = 36); ABCB (*n* = 32); ABHB (*n* = 43)

Developmental buffering suppresses variation during development. Hence we tested whether maternal buffering level and maternal embryonic thermal stress impact on the level of heterogeneity of the maternal mRNAs in the eggs. Our previous studies showed that the level of buffering against embryonic heat stress is maternally inherited in *Ciona* and is markedly greater in type A than in type B [17]. Hence cross hybridization of type A eggs to type B sperm produces higher level of embryonic developmental buffering than a conspecific cross of type B. We made a cross of type A eggs and type B sperm, and treated a batch of embryos by thermal stress and left a second batch unstressed, as we did with the conspecific cross of type B embryos, and reared the juveniles to mature adults in the marina (‘AB cross’; Fig. 3). We collected eggs from a heat-stressed individual (ABH) and an unstressed (control) individual (ABC), conducted single-egg transcriptome analyses, and compared CV^2^ between the two clutches using normalized transcript levels (TPM). We found an overall decrease of CV^2^ in the ABH clutch compared to the ABC clutch (mean value of log_10_(CV^2^H/CV^2^C) = -0.13) (Fig. 4b; ANCOVA test, estimate -0.0035, t-value -22.4, *P* < 2.2e^−16^), which was prominent compared to the decrease in BB (Fig. 4a). We also examined the genes that showed a five-fold increase or decrease in the value of CV^2^ between heat-shocked and control individuals (Fig. 4d, e), and compared those genes with those showing the same difference in the heterogeneity of expression in the type BB cross (Table S3). We found a trend of those genes increased variation in expression have lower expression levels than those genes decreased variation. In addition, those increased variation tend to decrease expression by maternal embryonic thermal stress, whereas those decreased variation tend to increase expression by maternal embryonic thermal stress. On the other hand, we found no genes in common between the clutches from BB and AB crosses that either increased or decreased in the value of CV^2^ five-fold. The data suggest that alteration of heterogeneity is controlled by the mother. We also made alternative crosses using type B eggs and type A sperm (‘BA’), and type A conspecific crosses (‘AA’). None of these crosses survived in this experiment.

### Intergenerational inheritance of heterogeneity in maternal mRNAs

We next hypothesized that, for the change in heterogeneity to have an evolutionary effect, any observed heterogeneity would need to be maintained in the next generation even in the absence of environmental stress. To test this, we collected eggs from an ABH adult and an ABC adult, crossed them with sperm from a single type BB individual that was caught in wild, and reared the resulting F2 progeny, without embryonic thermal shock, in the lab and then in the marina (‘ABHB’ and ‘ABCB’ respectively; Fig. 3). Once these F2 produced gametes, we collected eggs individually from a type ABHB adult and a type ABCB adult for transcriptome analysis. We found a large increase in CV^2^ value of the ABHB eggs (mean value of log_10_(CV^2^H/CV^2^C) = 0.20) (Fig. 4c; ANCOVA test, estimate 0.0040, t-value 25.5, *P* < 2.2e-16), showing that decrease of CV^2^ in the F2 generation was not inherited, but was in fact reversed. We also identified genes showing fivefold changes in CV^2^ (Fig. 4f, g) and tested whether the same outlier genes appeared in the clutches from the previous generation (comparison of AB and ABB generations). We found a surprisingly small number of common genes between the clutches. We found only two genes, PARP14 (poly(ADP-ribose) polymerase family member 14(KY2019:KY.Chr9.491)) and HSD17B14 (hydroxysteroid 17-beta dehydrogenase 14 (KY2019:KY.Chr2.1246), in which the value of CV^2^ was increased more than five-fold by thermal stress in both AB and ABB crosses. We also found crystalin alpha B (KY2019:KY.Chr3.484 and KY2019:KY.Chr3.485) and HSPA1B (heat shock protein family A (Hsp70) member 1B (KY2019:KY.Chr3.426), for which the value of CV^2^ decreased by five times by thermal stress in both generations.

We also tested which factors impacted on the homogeneity of variance in all the six sample sets: BBC, BBH, ABC, ABH, ABCB and ABHB. A Bartlett test for all the TPM values from these samples showed that embryonic thermal stress (*P* < 2.2E-16) and the type of cross (*P* < 2.2E-16) both affected the overall homogeneity of variance in the maternal mRNAs.

### Impact of thermal stress on maternally supplied developmental buffering molecules

We next tried to investigate what kind of functional properties correlate with the difference in variation in maternal mRNAs. However, in *Ciona intestinalis,* approximately 20% of gene models do not have orthologues to mammalian gene models [29], making the use of functional categories challenging. Therefore, rather than summarizing by functional categories, we decided to focus on testing whether thermal stress affects the expression of developmental buffering genes of offspring as previously hypothesized [20].

**Fig. 5:**
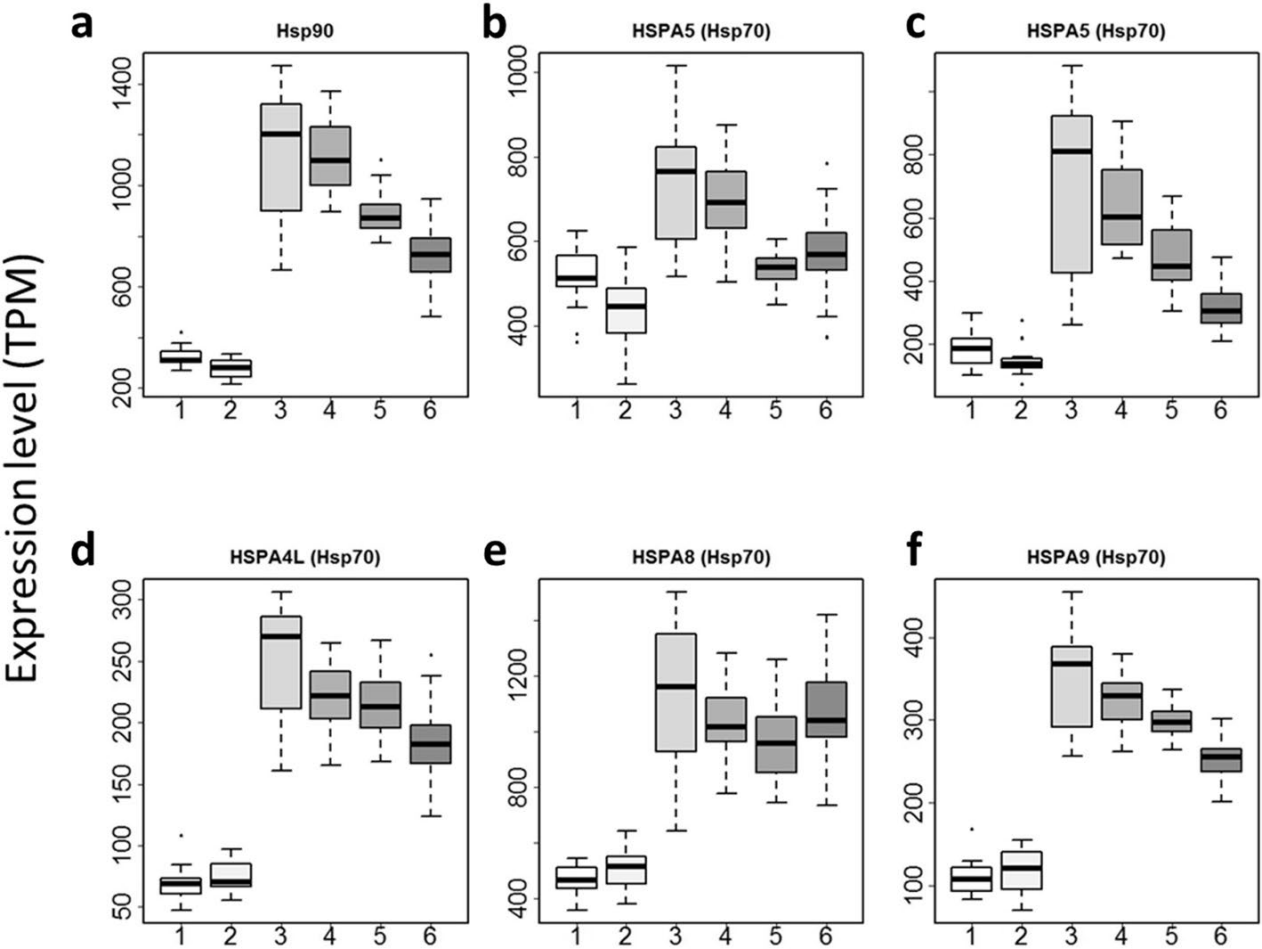
Impact of maternal embryonic thermal stress on maternally derived egg mRNAs encoding Hsp90 and Hsp70 family members. **a** Hsp90 (KY2019:KY.Chr1.1475). **b** HSPA5 (KY2019:KY.Chr9.923). **c** HSPA5 (KY2019:KY.Chr9.924). **d** HSPA4L (KY2019:KY.Chr14.131). **e** HSPA8 (KY2019:KY.Chr7.173). **f** HSPA9 (KY2019:KY.3.1407). 1, BBC (*n* = 14 eggs); 2, BBH (*n* = 16); 3, ABC (*n* = 37); 4, ABH (*n* = 36); 5, ABCB (*n* = 32); 6, ABHB (*n* = 43)

Previous studies on the impact of thermal stress on development largely focused on Hsp90 and Hsp70 chaperones (see review by Sato 2018 [20]), known as major players that minimize noise [30]. We found one gene putatively identified as a member of the Hsp90 family and five genes of the HSP70 family. Previous reports using *Caenorhabditis elegans* showed parental exposure to thermal stress increased expression level of Hsp90 [31], with transgenerational inheritance. However, we found that all the genes apart from one of the orthologues of HSPA5 and the orthologue of HSPA8 appeared to be downregulated in eggs in the two AB lineages as a result of maternal embryonic exposure to thermal stress, this included Hsp90 (Fig. 5). Apart from HSPA5 (KY2019:KY.Chr9.923) and HSPA9 (KY2019:KY.Chr3.1407), homogeneity of variance in these molecules is also significantly correlated to the genotype (Fig. 5; Table 1; Bartlett test, *P* < 8E-08), but not to thermal stress (Fig. 5; Table 1; Bartlett test, *P* > 0.01).

**Table 1:** *P*-values testing the impact of genotype and thermal stress and the interactions in the expression levels of Hsp90 and Hsp70 family members. In the variance tests (Bartlett test) *P* < 3.65E-06 was taken as significant after Bonferroni correction for multiple tests for 13,684 individual genes (shown in bold figures)

Gene ID (KY2019)	Gene_name	Bartlett_test (genotype)	Bartlett_test (genotype*stress)	Bartlett_test (stress)
KY.Chr1.1475	HSP90B1	**8.89E-16**	**9.42E-15**	0.737
KY.Chr9.923	HSPA5	0.03998	0.009039	0.3665
KY.Chr9.924	HSPA5	**8.81E-13**	**2.84E-11**	0.03394
KY.Chr14.131	HSPA4L	**2.07E-09**	**1.25E-07**	0.0178
KY.Chr7.173	HSPA8	**7.04E-08**	**1.72E-09**	0.07145
KY.Chr3.1407	HSPA9	9.27E-06	0.001276	0.06303

**Fig. 6:**
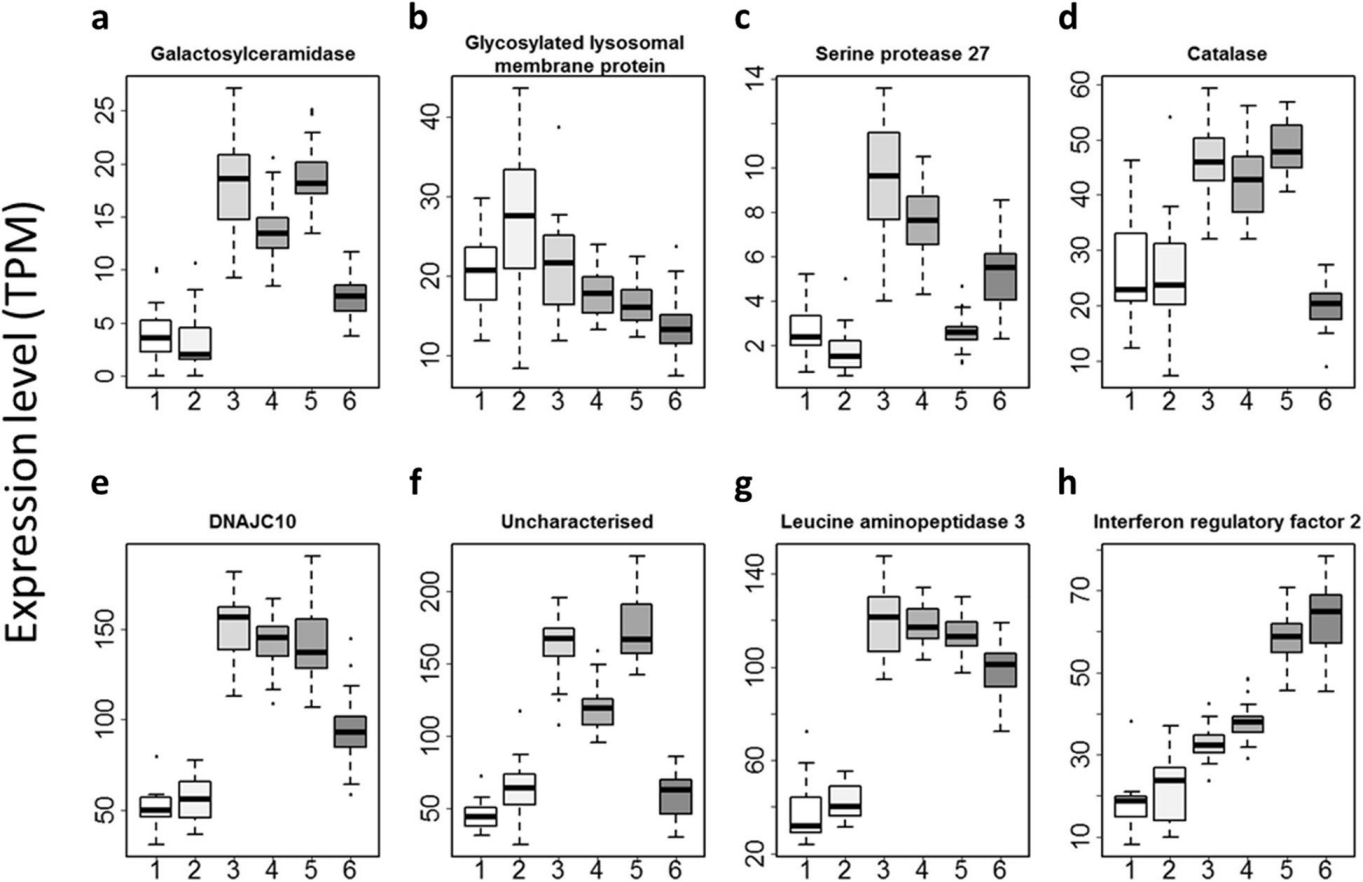
Impact of maternal embryonic thermal stress on maternal mRNAs of MDBGs in eggs. **a** Galactosylceramidase (KY2019:KY.Chr2.1046). **b** Glycosylated lysosomal membrane protein (KY2019:KY.Chr6.691). **c** Serine protease 27 (KY2019:KY.Chr7.688). **d** Catalase (KY2019:Chr2.2295). **e** DNAJC10 (KY2019:KY.Chr9.613). **f** an uncharacterised gene (KY2019:KY.8.888). **g** Leucine aminopeptidase 3 (LAP3) (KY2019:KY.2.1529). **h** Interferon regulatory factor 2 (KY2019:Chr14.407). 1, BBC (*n* = 14); 2, BBH (*n* = 16); 3, ABC (*n* = 37); 4, ABH (*n* = 36); 5, ABCB (*n* = 32); 6, ABHB (*n* = 43)

Other than Hsp90 and Hsp70 family members, our previous study identified 15 molecules that are maternally inherited developmental buffering molecules, which we named ‘maternally inherited developmental buffering genes (MDBGs)’ [25]. Out of the 15 MDBGs, we found eight in the transcriptome data we obtained in the present experiments. As we expected, genotype had a significant effect on the heterogeneity of gene expression of all of these genes (Fig. 6, Table 2, Table S3; *P* < 3E-06); apart from glycosylated lysosomal membrane protein (KY2019:KH.Chr6.691) there was up-regulation in type A egg-derived embryos relative to type B egg-derived embryos. It is of note that, in five of the eight genes, namely galactosylceramidase (KY2019:KY.Chr2.1046), catalase (KY2019:KY.Chr2.2295), DNAJC10 (KY2019:KY.Chr9.613), an uncharacterised gene (KY2019:KY.Chr8.888), and leucine aminopeptitdase 3 (KY2019:KY.Chr2.1529), expression levels were down-regulated in the eggs from thermally stressed mothers in all the BB, AB and ABB crosses (Fig. 6). We also tested whether genotype or embryonic thermal stress affected the variation of expression levels in MDBGs. In three of the genes, we found only genotype dependent differences in variation (Fig. 6; Table 2; Bartlett test, *P* < 2E-7), and in three of the eight genes we found a genotype dependent by thermal stress (Fig. 6; Table 2; Bartlett test, *P* < 2E-6). Importantly, however, we did not find any significant difference in variation solely as a result of thermal stress (Fig. 6; Table 2; Bartlett test, *P* > 1E-5). The data suggest that genotype, but not embryonic thermal stress solely, can impact on heterogeneity in MDBGs.

**Table 2:** *P*-values testing the impact of genotype, thermal stress, and their interaction in MDBG expression levels. In the variance tests (Bartlett test) *P* < 3.65E-06 was taken as significant after Bonferroni correction for multiple tests for 13,684 individual genes (shown in bold figures)

Gene ID (KY2019)	Gene_name	Bartlett_test (genotype)	Bartlett_test (genotype*stress)	Bartlett_test (stress)
KY.Chr2.1046	Galactosylceramidase	2.34E-04	0.5651	0.0008386
KY.Chr6.691	Glycosylated lysosomal membrane protein	0.0001767	1.31E-05	4.33E-02
KY.Chr7.688	Serine protease 27	**1.60E-07**	**3.08E-15**	1.06E-05
KY.Chr2.2295	Catalase	0.1261	**1.11E-07**	1.43E-01
KY.Chr9.613	DNAJC10	**6.07E-07**	**1.35E-06**	1.84E-01
KY.Chr8.888	Uncharacterised	**2.99E-07**	9.02E-06	3.11E-05
KY.Chr2.1529	Leucine aminopeptidase 3	0.08797	0.04342	0.16
KY.Chr14.407	Interferon regulatory factor 2	0.0001576	0.003725	0.5485

### Thermal stress had different effects on different signaling molecules

**Fig. 7:**
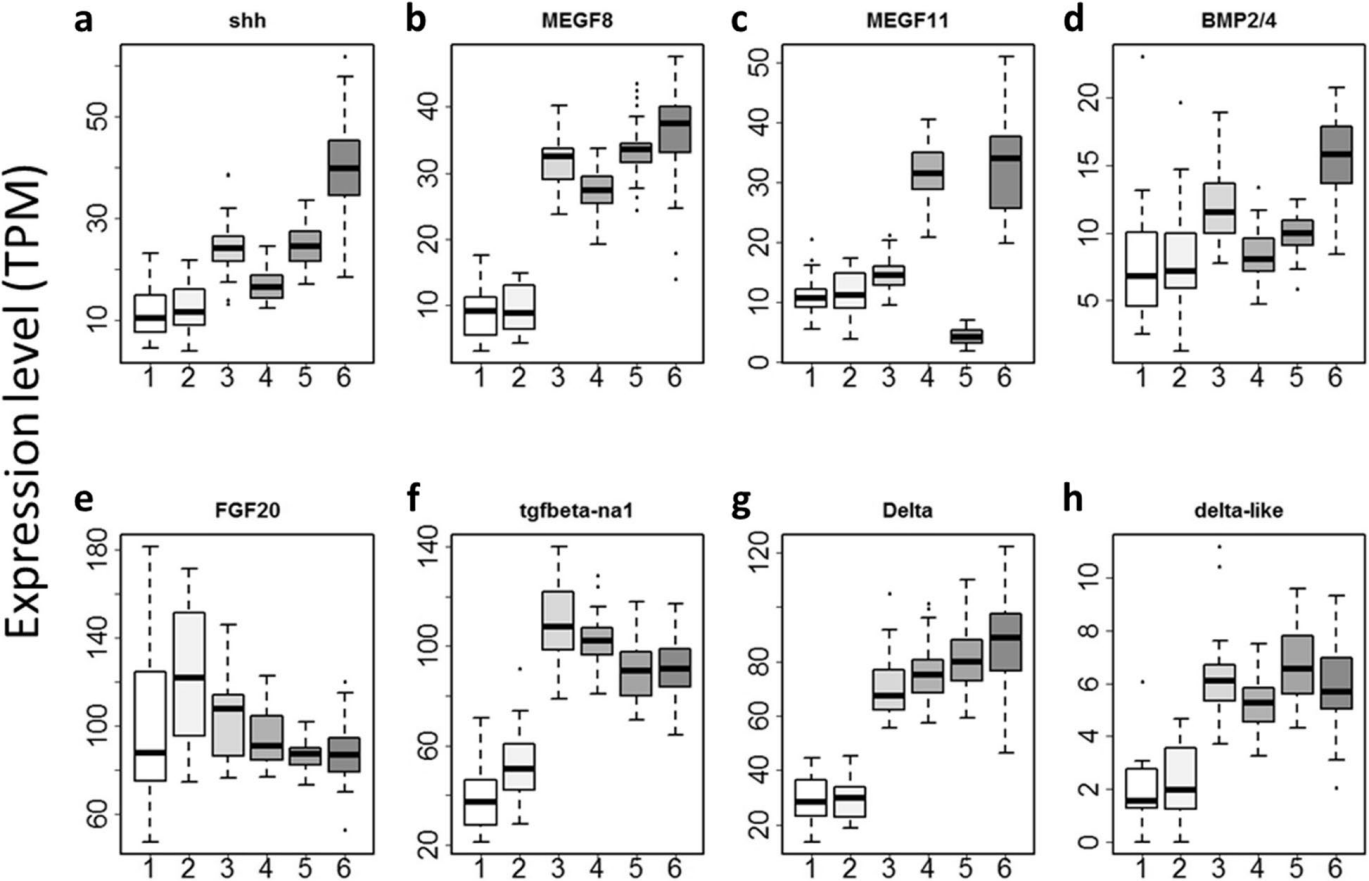
Impact of maternal embryonic thermal stress on various maternally supplied signaling molecules involved in development. **a ***shh* (KY2019.KY.Chr8.497). **b** MEGF8 (KY2019.KY.3.591). **c** MEGF11 (KY2019.KY.Chr6.138). **d ***bmp2/4* (KY2019.KY.Chr4.449). **e ***fgf20* (KY2019.KY.14.235). **f** TGF-β (KY2019.KY.Chr4.450). **g ***delta* (KY2019.KY.14.1158). **h ***delta-like* (KY2019.KY.11.146). 1, BBC (*n* = 14); 2, BBH (*n* = 16); 3, ABC (*n* = 37); 4, ABH (*n* = 36); 5, ABCB (*n* = 32); 6, ABHB (*n* = 43)

We also investigated how thermal stress potentially alters development by changes in expression of developmentally relevant genes. Previous studies suggested that stability of signaling molecules is the key to developmental buffering [25, 32], suggesting that differences in variation in the expression of major signaling molecules in development may affect the stability of early embryogenesis. To this end, we examined expression levels of major signaling molecules involved in development from the transcriptome datasets (Fig. 7; Table 3). We found eight signaling molecules in our datasets, namely *shh*, *bmp2/4*, TGF-β, *fgf20*, MEGF(multiple-EGF domain containing gene)8, MEGF11, *delta* and *delta-like*. Unlike Hsps and MDBGs, we found that a significant difference in the variance as caused by embryonic thermal stress in MEGF11 (Bartlett test, *P* < 6E-10) and *shh* (Bartlett test, *P* < 9E-9). In *shh*, *bmp2/4*, MEGF8 and *fgf20*, the effect of embryonic thermal stress on the homogeneity of variance was genotype dependent (Bartlett test, *P* < 3E-6; Table 3). In *fgf20* and MEGF11, heterogeneity of expression was significantly influenced by the genotype.

**Table 3:** *P*-values testing the impact of genotype and thermal stress, and the interactions in the expression levels of signaling molecules. In the variance tests (Bartlett test) *P* < 3.65E-06 was taken as significant after Bonferroni correction for multiple tests for 13,684 individual genes (shown in bold figures)

Gene ID (KY2019)	Gene_name	Bartlett_test (genotype)	Bartlett_test (genotype*stress)	Bartlett_test (stress)
KY.Chr8.497	*shh*	3.90E-05	**2.20E-16**	**5.20E-10**
KY.Chr4.449	*bmp2/4*	0.05176	**2.72E-06**	0.001363
KY.Chr4.450	*tgfb-na1*	0.3433	0.03976	0.005334
KY.Chr14.235	*fgf20*	**3.39E-14**	**2.83E-11**	6.18E-01
KY.Chr6.138	MEGF11	**5.03E-10**	0.02391	**8.08E-09**
KY.Chr3.591	MEGF8	0.002218	**1.72E-06**	0.3907
KY.Chr14.1158	*delta*	0.000457	0.00697	0.2083
KY.Chr11.146	*delta-like*	0.9771	0.9588	0.113

## Discussion

The biological conclusions drawn from this study are limited by a number of experimental constraints, namely the use of only one mother from each cross, single thermal stress regime was examined, and the number of reads we obtained from sequence analysis varied. However, based on this proof of concept study, we suggest that there are apparent differences in homogeneity of maternal mRNA provisioning in eggs produced by individuals exposed to thermal stress during their embryonic stage, but the type of effect was dependent on the particular genotype: eggs from a BB individual showed a slight decrease in variance, whereas eggs from an AB individual showed a clear overall decrease, and eggs from an ABB individual showed an overall large increase in variance following embryonic thermal stress. Furthermore, we found only a very limited number of genes showing the same trend of increase or decrease in heterogeneity between AB (F1) and ABB (F2) generations. A previous study at a single-gene level showed transgenerational increase of variation of *hsp16.2* gene expression in *Caenorhabditis elegans* after 1 h heat shock at 37°C [28]. However, in our experiments with *Ciona* thermal stress did not directly affect homogeneity of variation in Hsps and MDBGs, suggesting that variation in buffering level is determined by the genotype or the individual that produced the eggs, and is not modified by maternal embryonic thermal stress.

In contrast, we found a significant impact on the homogeneity of variance by embryonic thermal stress in two of the signaling molecules found in maternal mRNAs, *shh* and MEGF11. Moreover, each genotype reacted differently to embryonic thermal stress in terms of variance in *shh*, *bmp2/4*, *fgf20* and MEGF8. Of these, FGF signaling and BMP signaling are important in determining neural fate in chordates [33]. The late neurula to early tailbud stage at which we exposed the embryos to thermal stress is the stage when chordate characteristics such as notochord and neural tube appear. This stage is well conserved amongst chordates and less variable than other stages. Therefore, examining the impact of environmental stress at this embryonic stage on change in morphological variation at later stages is of interest in the light of how body plan evolved by environmental stressors. Our data suggest a possible route by which the epigenetic landscape could be altered by the maternal environment. Mechanisms by which de-stabilization of expression levels can be inherited warrant further study to better understand environmentally driven phenotypic evolution.

A number of examples have provided support for the ‘bet-hedging’ [6–9, 34, 35], in that between-sibling variation increases after parental exposure to environmental stress. However, our study did not support the hypothesis in the eggs of heat-treated mothers; the overall variation was decreased in both BB and AB, and only increased in the generation following AB, ABB, in which no new thermal shock was imposed. At a single-gene level, however, we observed that expression levels of several signaling molecules in type A-egg-lineages reacted more to thermal stress than they did in the type B-egg-lineage (Table 3; Fig. 7). Previous studies showed that development is well-buffered in type A egg-derived embryos [17], thus we predicted that the expression levels of signaling molecules would be less variable in type A-egg-derived lineages than in type B conspecific crosses. Of course, there are a number of factors to be considered in our experiments before testing any hypotheses with our data. The pattern of difference in variation in the maternal mRNAs might depend on the type of environmental stimulus and might differ between individual mothers. Previous work on the variation of maternal mRNAs in zebrafish showed that variation in maternal mRNA was tightly linked to the mother [36]. In addition, AB and ABB crosses might have epigenetic changes because of hybridization. These limitations may be overcome by investigating eggs from multiple individuals in various further crosses in the future.

How is provision of maternal mRNAs altered by maternal life experience? It could be that an epigenetic change occurs in the maternal genome in the egg as a result of parental experience [37], or that alteration of the maternal genome occurs in the egg by excision of transposons or tandem duplication [38] induced by thermal stress. An intriguing possibility that requires further investigation is RNA transport from parental tissues. Recent studies have shown paternal tissue can epigenetically control gene expression in embryos through transporting miRNA to the sperm [39–43]. In contrast to sperm, previous studies showed transcription of maternal genome in the egg is a major source of maternal mRNA in the egg [14] and observation of RNA transport from outside the egg to inside the egg is rather limited [44–46]. However, many observations have been made of changes in thermal tolerance [19] and behavior [47, 12] depending on parental experience, suggesting a possibility of RNA transfer to the egg in a number of different species in different taxa. A future challenge will be to elucidate the mechanisms altering maternal mRNAs in the egg depending on maternal life experience, and how it impacts on the variation of developmental phenotype.

## Conclusions

Our data suggest that genetic background and maternal embryonic thermal stress both affected overall homogeneity of maternal mRNA expression between individual eggs within a clutch. Whilst our data suggest that developmental buffering may not be affected by embryonic thermal stress, we observed that heterogeneity in the expression of several signaling molecules was affected by embryonic thermal stress. We suggest the possibility that embryonic environment modifies development of the next generation by altering heterogeneity in the maternal provisioning of signaling molecules. In addition to evolutionary implications, how organismal development is affected by maternal environment is one of the key issues to predict organismal development, health and disease, the so-called ‘genotype-to-phenotype map’ [47]. However, such study at a molecular level is still limited. Whilst the limited replication in this study calls for caution when interpreting these results, our study identifies a model system and experimental approach capable of providing a significant step forward in understanding the influence of maternal environment on developmental variation between siblings.

## Material and Methods

### Animals, eggs and embryos

Wild adults of *C. intestinalis* type A (*C. robusta*) and type B (*C. intestinalis*) were collected from either Queen Anne’s Battery or Sutton Harbour in Plymouth, UK, during summer (July and August) in 2017, and kept in tanks under identical conditions at 17ºC and continuous light for 1–2 days until dissection. Animals were fed a mixture of *Rhinomonas reticulata* and *Isochrysis galbana* once a day. Eggs and sperm were collected by dissection and fertilized as described previously [23, 48]. The allocations of the wild adults to Type A or B based on morphology were confirmed retrospectively by determining the genotype of four loci using sperm samples, as described by Sato et al. (2012) [23].

### Heat-shock experiments and common garden rearing

The experimental design is shown in Figs. 1 and 3. Briefly, we made four different combinations of crosses using two sibling species of *C. intestinalis*: We denote type A conspecific crosses as ‘AA’, type B conspecific crosses as ‘BB’, crosses using type A eggs and type B sperm as ‘AB’, and type AB eggs and type B sperm as type ‘ABB’. Approximately half of embryos from each cross were exposed to thermal stress at 26.5 °C for 1 h at 8 h post fertilization as previously described [17], and those batches in which > 80% hatched normally in control conditions were used for further analysis. Hatched larvae were kept in a plastic petri dish for three days until the larvae settled, metamorphosed and started feeding. We fed juveniles with a mixture of *Rhinomonas reticulata* and *Isochrysis galbana* twice a day for a week and transferred them to Queen Anne’s Battery Marina for common-garden rearing under field conditions as described by Sato et al. (2012) [23] and Sato et al. (2014) [48]. Whilst lab conditions were kept relatively constant, rearing periods of the different crosses and generations in the marina were necessarily non-concurrent, and thus incorporated natural variation in ambient conditions. When the animals were mature, we dissected eggs and sperm from oviduct and spermiduct, respectively. All the detail of crosses used are summarized in Table S1.

### Single egg sorting and cDNA library construction

Dechorionated eggs from an adult heat-treated BB (‘BBH’) individual and an adult control BB (‘BBC’) individual were dissected from the oviduct and kept on ice, collected individually in a drop of 2.3 µl of filtered sea water using 10 µl pipettes, mixed with lysis solution containing 0.1–1% TritonX (Sigma) and 2U Recombinant RNase inhibitor (Clontech) and kept at -80 °C before being processed for cDNA library construction. Dechorionated eggs from an adult from heat treated AB, an adult from control AB, an adult from heat treated ABB (‘ABHB’) and an adult from control ABB (‘ABCB’) were stored in RNA Later and kept at -80 °C before being manually isolated similarly to type BB eggs and processed for cDNA library construction. cDNA library construction was conducted following instructions of Picelli et al. (2014) [49], with some modifications: We used 0.016 µl of 1:100,000 External RNA Controls Consortium (ERCC) mRNA spike-in dilution for 20 µl total volume of cDNAs for each egg in BBC and BBH, whereas we used 0.29 µl of 1:1000 ERCC mRNA spike-in dilution for 20 µl total volume of cDNAs for each egg from ABC, ABH, ABCB and ABHB. Amplification involved 10 cycles of reverse transcription (step 11) gave the best results in amplification of cDNAs among 10–13 cycles. We also used 11 cycles of PCR re-amplification for BB samples and 14 cycles for AB and ABB (step 14), which gave us enough amount of DNA for sequencing. 21–23 million reads per sample were obtained and processed for analysis.

### Transcriptome sequencing analysis

We obtained 1.7–2.2 million reads (average 1.9 million reads) for BBC, 1.7–2.4 million reads (average 1.8 million reads) for BBH, and 5.9–77 million reads (average 29 million reads) for ABC, 7.7–103 million reads (average 29 million reads) for ABH, 2.6–114 million reads (average 32 million reads) for ABCB, and 12–106 million reads (average 34 million reads) for ABHB. The adaptor sequences were trimmed from all reads using Trimmomatic [50], and trimmed reads > 10 bp in length were kept. Trimmed paired reads were mapped onto the HT *Ciona intestinalis* type A genome [51] using robust mapping software, BWA mem [52], using the default settings. Differential gene expression was calculated by featureCounts [53] using two gtf files: one including all the gene ids in KY gene models [51] except for 45 s rDNA, and the other containing only 45 s rDNA.

To calculate the variation in expression level of maternal mRNAs, we normalized the total read counts by TPM normalization of the gene models that showed expression in over 50% of samples (Table S2) and calculated squared coefficients of variance (CV^2^) using R script [54]. We also excluded data from single eggs producing low-quality data (almost no gene showing any expression). To exclude the data from single eggs with low coverage, we used only those gene models that had values above zero for over 20% of all the single-egg transcriptome samples.

### Supplementary Information

**Additional file 1: Table S1.** Frequency of normal larvae after heat shock of the parents.

**Additional file 2: Table S2.** TPM values of all samples used for analysis (provided as a separate excel sheet).

**Additional file 3: Table S3.** Average, variance and CV^2^ for BB, AB, and ABB datasets (provided as a separate excel sheet). These genes for which the value of CV^2^ increased or decreased by five times by thermal stress are shown as '1' in '_P5' or '_M5' column, respectively.

## Data Availability

Raw sequence data: Deposited to DDBJ Sequence Read Archive under the accession numbers DRX325090-DRX325279 and DRR493923-DRR493952. Code: binbucket.org/atsukos/Heterogeneity of maternal mRNAs.
