# Supplementary material for: Heterogeneity in maternal mRNAs within clutches of eggs in response to thermal stress during the embryonic stage
Source: BMC Ecol Evol. 2024 Feb 12;24:21. doi: 10.1186/s12862-024-02203-8 (PMC10860308; doi:10.1186/s12862-024-02203-8)
Supplement: Supplementary file 1 — Additional file 1: Table S1. Frequency of normal larvae after heat shock of the parents. [file 12862_2024_2203_MOESM1_ESM.docx]

Heterogeneity in maternal mRNAs within clutches of eggs in response to thermal stress during the embryonic stage

Atsuko Sato^1, 2, 5, 6^*, Yukie Mihirogi^1^, Christine Wood^2^, Yutaka Suzuki^3^, Manuela Truebano^4^, John Bishop^2^

Supplementary Tables

Table S1 Frequency of normal larvae after heat shock of the parents

Table S2 TPM values of all samples used for analysis (provided as a separate excel sheet)

Table S3 Average, variance and CV^2^ for BB, AB, and ABB datasets (provided as a separate excel sheet)

Table S1 **Buffering levels of the samples used**.

| **Egg ID** | **Genotype** | **Egg P mother** | **Egg P father** | **Egg generation** | **Hatch** | **Frequency of normal larvae** |
| --- | --- | --- | --- | --- | --- | --- |
| 15 | BBC | 2017-11 | 2017-12 | F1 | 0.8189 | 0.9947 |
| 14 | BBH | 2017-11 | 2017-12 | F1 | 0.8421 | 0.9453 |
| 7 | ABC | 2017-14 | 2017-16 | F1 | 1 | 0.9677 |
| 9 | ABH | 2017-14 | 2017-16 | F1 | 0.9361 | 0.9469 |
| 44 | ABCB | 2017_14  x 2017_16 control | 2017_4  x 2017_3 control | F2 | 0.9038 | 0.8617 |
| 47 | ABHB | 2017_14  x 2017_16 heat | 2017_4  x 2017_3 control | F2 | 0.9649 | 0.9818 |
